# Editorial: The ethics of speech ownership in the context of neural control of augmented assistive communication

**DOI:** 10.3389/fnhum.2024.1468938

**Published:** 2024-08-06

**Authors:** Zachary Freudenburg, Julia Berezutskaya, Cornelia Herbert

**Affiliations:** ^1^Brain Center, University Medical Center Utrecht, Utrecht, Netherlands; ^2^Applied Emotion and Motivation Psychology, Institute of Psychology and Education, Ulm University, Ulm, Germany

**Keywords:** Brain-Computer Interfaces (BCI), augmented assistive communication (AAC), speech ownership, user agency, executory control, guidance control

## 1 Introduction

This Research Topic focuses on the complex and unique ethical considerations and design challenges with respect to preserving user agency (the reflection of user intention in system performance) when augmented assistive communication (AAC) devices are controlled via neural signals. Such devices represent a special category of Brain-Computer Interfaces (BCIs) and AACs because they involved both direct sensing and interpretation of brain activity and assistive communication. The ethical discussions around BCIs and AACs fully apply Speech-BCIs (BCIs that produce speech content). However, when the challenges of interpreting neuronal signals intersect with AAC device imposed limitations on user expression unique issues arise. In addition, the recent growth in the technical capabilities of BCIs is driving a rapid expansion in possible use-cases for Speech-BCIs. Hence, this Research Topic aims to further and sharpen the discussion of ensuring user agency and accessing speech ownership in the context of Speech-BCIs at a time when key design decisions that can greatly influence these issues are still being made.

## 2 Main themes and topics

This discussion is explored in three original manuscripts by leading ethicists and researchers at the cutting edge of the Speech-BCI field, complemented by two reprinted works that help frame the context. Three central themes emerge:

1) **Speech-BCIs represent shared control systems** where users control cognitive activities and complex AI systems decoded brain signal features and infer intended speech output.
2) A clear **definition of the unique ethical concerns and terms of the Speech-BCI field** is needed to make the design challenges concrete. A clear definition of the unique ethical concerns and terms of the Speech-BCI field
3) The design choices to promote agency often **weigh performance and speed against transparency of speech ownership**.

A strong argument can be made that the improvements in speed and correctness facilitated by AIs such as Large Language Models (LLMs) offer increased user agency by expanding the user's ability to communicate. However, AI assistance adds an interpretive layer with potential biases on top of the often imperfect speech content directly decoded from neural signals. All three original works touch on this as a central tension point at the heart of the ethical discussions in the field.

## 3 Summary of original manuscripts

First, a narrative review of ethical issues prominent in Speech-BCI focused literature intended to build a framework for discussion of ethical design recommendations is given in van Stuijvenberg et al.. The concepts of designing for **executory control** [if and when speech is externalized (Maslen and Rainey, 2021)] vs. **guidance control** [the shaping of how speech is formulated (Sankaran et al.)] that are emerging as guiding design principals in Speech-BCI literature are introduced. They also provide suggestions for clarifying the terminology used when discussing the ethics of Speech-BCI design decisions. They emphasize the importance of defining whether a Speech-BCI is used as an **instrumental tool**, for communicating simple messages, for example, about care needs and/or as an **expressive tool**, for communicating more complex opinions and emotions. When used as an instrumental tool, accuracy and transparency of meaning may be prioritized over speed and naturalness. Whereas, when used as an expressive tool, speed and fluency of language may be more relevant performance goals. While natural language is clearly used for both, Speech-BCIs are imperfect communication tools and clarifying their intended use sharpens ethical discussions. In addition**, there is a clear difference in the context of Speech-BCIs between speech that is formalized internally, but not intended for external communication and speech meant to be externalized by the BCI user**. However, the terminology used to describe this distinction has not been standardized in the field, potentially leading to misunderstandings, especially when communicating to the broader public.

The second original manuscript by Rainey broadens the discussion by pointing out that effective **Speech-BCI use will be a learned skill**. As such, users will need to learn to work with or around the system's limitations. Rainey discusses ways in which this skilled use can lead to a disconnect between the face value meaning of the produced speech and the users intended meaning even when executory and guidance control have been established. Rainey explores the ethical consequences a **“reasons-responsiveness”** [the “relationship between human reasons and the behavior of systems which include human and nonhuman agents” (Mecacci and Santoni de Sio, 2020)] ambiguity can have for the attribution of ownership of BCI-produced speech. This expands upon the issue of speech ownership when control over speech output is imperfect and/or shared as presented above.

Finally, a perspective of researchers at the forefront of Speech-BCIs use by people with severe speech disabilities is given by Sankaran et al.. They discuss practical strategies for executive control by **allowing the user to review speech output before it is shared**, also suggested by van Stuijvenberg et al., and guidance control such as **allowing users to choose language models with specific biases** such as formal vs. informal speech.

## 4 Broader context

Additionally, two reprints provide a larger context for the discussion. A comparative review by Ishida et al. of the neuroethical issues represented in neuroethical and neuroscience journals stresses that both fields could benefit from integrating ethicists into research groups. Original research by Muncke et al. into the neural correlates of speech recognition in noisy environments highlights how context can effect speech-BCIs. A detailed discussion of the role human factors such as user mental states and traits like language competence and cultural background can have on BCI performance is given in a complementary Research Topic: *Analyzing and computing humans – the role of language, culture, brain and health*.

## 5 Conclusion and outlook

This Research Topic provides a basis to expand the discussion of the unique ethical issues inherent to direct neural control of AACs at a time when the exposure of Speech-BCIs in the media is rapidly increasing. As the public watches these devices transition from exciting concept to clinical reality it is critical to have clear discussion of the ethical implications of design decisions making this transition possible to avoid misconceptions about the aims and limitations of Speech-BCIs with respect to speech ownership that could jeopardize their acceptance.

## Author contributions

ZF: Conceptualization, Writing – original draft, Writing – review & editing. JB: Writing – review & editing. CH: Writing – review & editing.
